# Controversy

**Published:** 1872-09

**Authors:** 


### CONTROVERSY.

Our readers will see that much of our editorial space has been taken in reply to Dr. Gaillard's strictures upon the Senior Editor of this Journal. If this controversy could have been avoided, by anything we could have done, they would never have been bored by it; but private character and the standing of THE COMPANION was at stake, and hence, reluctantly, we have been forced to combat an enemy whose motive, it seems, is to injure, to the utmost, every one and everything connected with our journal.

We trust, however, true principles have been evolved in this controversy, called forth by the unprovoked assults of Dr. Gaillard. And to that extent, we hope our readers have been profited.

